# Exploring *Tetraselmis chui* microbiomes—functional metagenomics for novel catalases and superoxide dismutases

**DOI:** 10.1007/s00253-024-13395-w

**Published:** 2025-01-13

**Authors:** Jascha F. H. Macdonald, Yuchen Han, Yekaterina Astafyeva, Lutgardis Bergmann, Marno Gurschke, Philipp Dirksen, Patrick Blümke, Yannik K. H. Schneider, Malik Alawi, Sebastian Lippemeier, Jeanette H. Andersen, Ines Krohn

**Affiliations:** 1 Department of Microbiology and Biotechnology, Institute of Plant Science and Microbiology, University of Hamburg, Ohnhorststr.18, 22609 Hamburg, Germany; 2 Bioinformatics Core, University Medical Center Hamburg-Eppendorf, Hamburg, Germany; 3 Leibniz Institute of Virology, Hamburg, Germany; 4 Marbio, Faculty of Biosciences, Fisheries and Economics, UiT—The Arctic University of Norway, Tromsø, Norway; 5 BlueBioTech GmbH, Büsum, Germany

**Keywords:** Antioxidants, Healthcare, Microalgae microbiome, *Tetraselmis chui*, Catalase, Superoxide dismutase, Metagenome

## Abstract

**Abstract:**

The focus on microalgae for applications in several fields, e.g. resources for biofuel, the food industry, cosmetics, nutraceuticals, biotechnology, and healthcare, has gained increasing attention over the last decades. In this study, we investigate the microbiome of the cultured microalga *Tetraselmis chui* (*T. chui*) to highlight their potential for health benefits. In this context, biomolecules like antioxidants play a crucial role in the well-being of living organisms as they metabolise harmful reactive oxygen species (ROS) to reduce oxidative stress. Impaired processing of ROS leads to damaged cells and increases the risk of cancer, inflammatory diseases, and diabetes, among others. Here, we identify, characterise, and test bacterial antioxidants derived from the *T. chui* microbiome metagenome dataset. We identified 258 genes coding for proteins with potential antioxidant activity. Of those, four novel enzymes are expressed and identified as two superoxide dismutases (SOD), TcJM_SOD2 and TcIK_SOD3, and two catalases (CAT), TcJM_CAT2 and TcIK_CAT3. Extensive analyses characterised all implemented enzymes as active even in concentrations down to 25 ng*ml^−1^ for the SODs and 15 ng*ml^−1^ for the CATs. Furthermore, sequence-based analyses assign TcJM_SOD2 and TcIK_SOD3 to iron superoxide dismutases (Fe SODs) and TcJM_CAT2 and TcIK_CAT3 to heme-containing catalases. These candidates are phylogenetically classified within the phylum Pseudomonadota. Regarding the biotechnological potential, a toxicity assay did not indicate any harmful effects. The introduced enzymes may benefit medical applications and expand the potential of microalgae microbiomes.

**Key points:**

*• Omics-based discoveries of antioxidant enzymes from Tetraselmis chui microbiome*

*• Two superoxide dismutases and two catalases are identified and tested for activity*

*• Enzyme sensitivity highlights biotechnological potential of microalgae microbiomes*

**Supplementary Information:**

The online version contains supplementary material available at 10.1007/s00253-024-13395-w.

## Introduction

Microalgae like *Tetraselmis chui* have garnered significant attention due to their potential applications in various fields, including biotechnology, biofuel production, food as well as nutraceutical additives, and environmental remediation (Grierson et al. 2011; Cerezuela et al. 2012; Moser et al. 2022; Segovia-Campos et al. 2024). Large-scale farming of *T. chui* is already underway and being optimised through ongoing investigations (Khatoon et al. 2018; Coleman et al. 2023; Yusuf et al. 2023). *T. chui* is prominently used in food as protein enrichment and replacement product with nutritional benefits (Qazi et al. 2021). Most interestingly, *T. chui* cultures can be also applied to benefit environmental health by cleaning wastewater of fish farms (Villar-Navarro et al. 2022). Current research focusses on the processing and application of the algae in cosmetics and as animal feed, e.g. in fish farms (Sørensen et al. 2023; Garcia et al. 2024; Simon et al. 2024;). 

Overall, *T. chui* is a species of green microalgae belonging to a genus of primary producers in marine and brackish water environments. One area of interest regarding *T. chui* and other microalgae is their interaction with the environmental microbiome, particularly in aquatic ecosystems. The microbiome refers to the complex community of microorganisms, including bacteria, fungi, and protists, that inhabit a specific environment. These microorganisms form symbiotic relationships that play a crucial role in nutrient cycling, carbon fixation, and the overall dynamics of the ecosystem (Cirri and Pohnert 2019).

Molecular tools like functional metagenomic analyses enable us to screen microbiomes to investigate those interactions and screen for novel, valuable biomolecules. For example, antioxidants play a crucial role in mitigating oxidative stress, which can result from various environmental factors such as exposure to pollutants, UV radiation, or fluctuations in temperature (Pizzino et al. 2017). Protection against free radicals like reactive oxygen species (ROS) is crucial for all living species, from single-cell organisms to mammals. Until today, several molecules acting as antioxidants are known, with superoxide dismutase (SOD) and catalase (CAT) among them.

Superoxide dismutases (E.C. 1.15.1.1) are divided into four groups according to their involved metal cofactors. They are indicated by the designation of Ni-SOD, Cu/Zn-SOD, Mn-SOD, and Fe-SOD, with Nickel, Copper/Zinc, Manganese, or Iron as cofactor, respectively. While Mn-SOD and Fe-SOD developed in prokaryotes, Cu/Zn-SOD occurred in eukaryotes (Steinman 1978; Smith and Doolittle 1992). Interestingly, Mn-SOD is found in eukaryote mitochondria, and Cu/Zn-SOD is found in pathogenic bacteria, contrary to their evolution in eukaryotes. Lastly, Ni-SOD is found in Streptomycetota and Cyanobacteria. Overall, SODs are found in all phylogenetic kingdoms (Abreu and Cabelli 2010).

Catalases are enzymes that catalyse the decomposition of hydrogen peroxide into water and oxygen, thereby protecting cells from oxidative damage (Chelikani et al. 2004). CATs belong to an abundant group of heme-containing enzymes. These enzymes found in bacteria, Eukarya and Archaea are highly active. The heme-containing CATs can be classified into two major groups: monofunctional CATs and bifunctional catalase-peroxidases (Zamocky et al. 2008). The monofunctional CATs (EC 1.11.1.6) are divided into three clades, which evolved early in the development of the gene family through gene duplication events (Klotz 2003; Zamocky et al. 2008). They belong to the hydrogen peroxide reductases. The bifunctional catalase-peroxidase (EC 1.11.1.7) is a donor oxidoreductase that also exhibits peroxidase activity. Additionally, Mn-CATs belong to a third group and form the nonheme manganese-containing CATs (EC 1.11.1.6). This minor group is only found in bacteria (Zamocky et al. 2008; Savelli et al. 2019).

Recent studies on *T. chui* and the associated microbiome have shown that these microalgae can produce antioxidant enzymes like SODs and CATs as part of their defence mechanisms against oxidative stress (Widowati et al. 2017). Additionally, interactions between microalgae and bacteria within the microbiome can influence the production and activity of antioxidants. For example, certain bacterial species may produce compounds that stimulate the production of antioxidants in microalgae or provide protection against oxidative stress through other mechanisms (Krohn et al. 2022). The potential of *Tetraselmis* species in health applications has already been described in former studies (Sansone et al. 2017; Moser et al. 2022; Lopes et al. 2024). Additionally, as this genus and its species *T. chui* have already been applied in culturing approaches (Day and Fenwick 1993; Lu et al. 2017; Patrinou et al. 2022), we choose the microbiome of this algae as the foundation for the metagenome analyses.

Understanding the relationship between *T. chui*, the microbiome, and antioxidants has implications for various applications, including aquaculture, bioremediation, and the development of novel antioxidant products. By elucidating the mechanisms underlying these interactions, researchers can harness the beneficial properties of microalgae and their associated microbiota for environmental and biotechnological purposes. This study investigates *T. chui* culture and their associated microbiome to implement novel and efficient antioxidant enzymes.

## Material and methods

### Culture conditions of the microalgae *T. chui* including associated microorganisms

Single microalgae cultures of *T. chui* SAG 8–6 were cultivated at 21 °C with natural light intensity (14 h light period per day, 10 h dark period per day) in liquid F-medium, simulating its native environmental habitat to support optimal growth and metabolic activity (Guillard and Ryther 1962). Cultures were renewed in 2-month intervals by the inoculation of fresh medium.

### Metagenome and total DNA isolation

Cells of a *T. chui* SAG 8–6 culture, including the associated microbial community, were used to prepare the metagenome dataset. For this, 30 ml of the culture was centrifuged for 30 min at 5000 × g. The resulting pellet was resuspended in the Elution Buffer provided by NucleoBond High Molecular Weight Genomic DNA-Kit (MACHEREY‑NAGEL, Düren, Germany). The microalgae suspension was shock-frozen in liquid nitrogen and, after thawing, pipetted on bashing beads. Forty-microlitre Buffer MG, 0.5 mg proteinase K, and 5 mg of lysozyme were added before it was bead-bashed on Vortex-Genie®2 (Scientific Industries, New York, NY, USA) four times for 1 min with breaks on ice for 30 s. After the addition of 600 ml Buffer MG, the suspension was incubated at 55 °C for 30 min in static conditions. DNA elution was performed according to the manufacturer’s instructions. Finally, DNA was eluted in 30 µl of deionised water. DNA concentration and purity were analysed using a NanoPhotometer® NP80 (IMPLEN, Westlake Village, CA, USA).

### Sequencing and assembly

Library preparation and sequencing were performed at the Leibniz Institute of Virology (LIV, Hamburg). One nanogram of DNA was subjected to library preparation with the Nextera XT DNA Library Preparation Kit (Illumina, product number: FC-131–1096) according to the manufacturer’s instructions. Illumina short-read sequencing was done on a NextSeq 500 (Illumina, San Diego, CA; USA) sequencer (NextSeq 500/550 High Output Kit v2.5, 300 Cycles, product number: 20022408) with 2 × 151 bp (plus 2 × 8 bp Index reads). Demultiplexing with bcl2fastq (default settings) yielded 86.6 mio reads of the *T. chui* metagenome sample.

Sequence reads were processed with fastp (v0.21.0) to remove sequences originating from sequencing adapters (Chen et al. 2018). Processed reads shorter than 40 bp were discarded. The remaining reads were assembled using IBDA-UD (v1.1.3) (Peng et al. 2012).

### Metagenomic dataset analyses and identification of novel antioxidants from *T. chui* microbiome

Critical features of antioxidant activity of *T. chui* microbiome were investigated using IMG function search. Data is shown in the total number of hits for possible antioxidant activity (Nordberg et al. 2014; Chen et al. 2019; Krohn et al. 2022). Genes were selected based on previous literature that investigated antioxidants such as SODs, rhodanese-related sulfurtransferase, CATs, peroxidase I, ferritin, glutaredoxin, glutathione peroxidase, cytochrome c peroxidase, alkylhydroperoxidase, deferrochelatase/peroxidase, and peroxiredoxin (Fones and Preston 2012; Buonvino et al. 2022; Ferdous and Yusof 2021; Matamoros et al. 2010; Lauritano et al. 2023).

Primers were designed from the reference gene in the metagenome for further cloning (Table 1). SignalP v.6.0 (Teufel et al. 2022) for N-terminal secretion signal prediction was conducted on the resulting amino sequence to exclude signal peptides from the final enzyme. In our study, we focus on intracellular antioxidant enzymes. Our sequenced-based analyses did not show any predicted signal peptide for all four characterised enzymes. Sequence-based protein structure predictions were performed using Alphafold2 and visualised with UCSF Chimera v.1.17.3 (Pettersen et al. 2004; Jumper et al. 2021). The sequences of SODs were compared with the non-redundant protein database of NCBI by using BLASTP. The sequences of bacterial catalases and peroxidases were extracted from RedoxiBase (Savelli et al. 2019, https://peroxibase.toulouse.inra.fr/). The phylogenetic tree was constructed with MEGA11 (Tamura et al. 2021) based on the Neighbor-joining method and JTT matrix-based model (Jones et al. 1992) with 1000 bootstrap replications after multiple alignments with T-Coffee (Notredame et al. 2000).

**Table 1:** Cloned antioxidant enzymes including function, length, primer design, IMG accession no., and phylogenetic assignment

***Enzyme***	*Function*	*Length (aa)*	*Primer fw (restriction site)*	*Primer rv (restriction site)*	*Source*	*IMG accession no. (Ga0499797_)*	*NCBI nucleotide-blast (perc. identity)*
*TcJM_SOD2*	Superoxide dismutase	199	CGC**GGATCC**ATGGCTTTTGAACTTCCCGAT (BamHI)	CCG**CTCGAG**CATGCGCGACGCGACG (XhoI)	*T. chui* microbiome	000027_110175_110774	*Pacificitalea manganoxidans* (88.9)
*TcIK_SOD3*	Superoxide dismutase	200	CGC**GGATCC**ATGGCTTTTGAACTTCCCGAT (BamHI)	CCC**AAGCTT**GCTTGTCGCAGCCTCAT (HindIII)	*T. chui* microbiome	000031_37060_37662	*Roseitalea porphyridii* (88.8)
*TcJM_CAT2*	Catalase	506	CGC**GGATCC**ATGACACGTCGCAAAGACAC (BamHI)	CCC**AAGCTT**GATTCCACTGATACTCTCGGCT (HindIII)	*T. chui* microbiome	000030_56127_57647	*Pseudosulfitobacter pseudonitzschiae* (99.6)
*TcIK_CAT3*	Catalase	484	CGC**GGATCC**ATGACCAAGGACTCAGGAAAACC (BamHI)	CCG**CTCGAG**GCCTTCCCTGACGCCC (XhoI)	*T. chui* microbiome	000173_13486_14940	*Roseovarius* sp. (87.6)

### Protein similarity network construction

ORFs in the metagenome of *T. chui* were annotated with Prodigal 2.6.3 in anonymous mode (Hyatt et al. 2010). The identified proteins were screened with hmmsearch 3.3.2 (hmmer.org) for the PFAM motifs PF00199 and PF06628 (Catalase), or PF00081 and PF02777 (Superoxide dismutase) using each motif’s trusted cutoff as threshold. Domain-containing proteins were aligned to the NCBI nr database (as of May 2024) with diamond v2.1.9.163 in BLASTP mode for taxonomic classification (Buchfink et al. 2021). Finally, pairwise similarities of these proteins were derived from an all-vs-all alignment and used for network visualisation. Structure comparisons were performed using the Dali server with entries of the RCBS protein database (PDB) (Berman 2000; Holm 2020).

### Cloning and purification of antioxidants

In order to express SOD candidates (TcJM_SOD2 and TcIK_SOD3) and CAT candidates (TcJM_CAT2 and TcIK_CAT) identified from the *T. chui* microbiome, gene fragments were amplified from the metagenomic DNA by PCR with the designed primers (Table 1). Two restriction sites at both ends of each PCR product were introduced by primers (Table 1, marked in bold). The restricted PCR fragment was ligated to the appropriate restricted and linearised vector pET21a( +) upstream of the His-tag sequence. After verification via sequencing, the final construct was heat-shock transformed into a chemically competent *Escherichia coli* Rosetta-gami 2(DE3) expression host. Overexpression was archived in autoinduction medium ZYM5052 (Studier 2005), including 100 µg*ml^−1^ ampicillin at 37 °C until an OD600 nm of 0.6 was reached, followed by an overnight cultivation at 22 °C. The enzymes were purified using Protino® Ni–NTA columns (Macherey–Nagel, Düren, Germany) following the protocol of polyhistidine-tagged proteins. Finally, the concentrated enzymes were rebuffered in 0.1 M Potassium-Phosphate Buffer (PPB) at pH 7 using Sartorius Vivaspin columns (Sartorius, Göttingen, Germany) and stored at 4 °C.

### Superoxide dismutase activity assay

The SOD Determination Kit (19160; Sigma-Aldrich, St. Louis, MO, USA) was used to determine the activity of the cloned, expressed, and purified superoxide dismutases (Huang et al. 2000). The assay uses the reaction of tetrazolium salt, which is reduced by superoxide anions to form a water-soluble formazan dye. This reaction is controlled by a xanthine oxidase and inhibited by the tested SODs. A higher activity of a putative SOD results in a higher inhibition of the described reaction. The reaction took place at 37 °C for 20 min. The inhibition rate was quantified according to the absorption of the tetrazolium salt at 440 nm.

Initial assays were performed to detect suitable enzyme concentrations for activity measurements by conducting the assay with decreasing enzyme concentrations until reaching results within the prescribed OD limits according to the manufacture’s protocol. Finally, 3000 ng*ml^−1^ of the enzyme stock in 0.1 M PPB pH 7 was tested in triplicates following the assay protocol. Simultaneously, tenfold higher and lower concentrations were tested as well. Assays with PPB served as control. The final assay volume was 240 µl with 20 µl of the enzyme stock. This resulted in the final tested enzyme assay concentrations of 2500 ng*ml^−1^, 250 ng*ml^−1^, and 25 ng*ml^−1^.

The results were tested for normal distribution using the Shapiro–Wilk test to ensure the data met the assumptions of parametric analysis (Shapiro and Wilk 1965). Subsequently, datasets following a normal distribution were compared using a two-tailed unpaired *t* test to evaluate differences between groups. Statistical significance was set at *p* < 0.05, and results are reported as mean ± standard deviation (SD). Analyses were performed using software R, ensuring robust statistical evaluation (R Core Team 2020).

### Catalase activity assay

Catalase activity was measured with the Catalase Colorimetric Activity Kit (EIACATC; Invitrogen, Carlsbad, CA, USA) (Croft et al. 2023). In this assay, horseradish peroxidase reacts with a provided substrate in the presence of H_2_O_2_, which creates a measurable colour shift of the colourimetry detection reagent. The reaction of the H_2_O_2_ with the tested catalases inhibits this colour shift and can be determined with absorption at 560 nm. Inhibition increases proportionally with the enzyme activity of the tested catalase. The substrate processing step lasted for 15 min at 20 °C. The enzyme Unit (U) is given in µmol*min^−1^. The enzyme activity U*ml^−1^ defines the quantity of substrate which is degraded per volume of the tested enzyme per min. This assay provided a 100 U*ml^−1^ bovine standard catalase to adjust the measured absorbance values at 560 nm to a resulting standard curve of y =  − 0.0625x + 0.3963 (*n* = 18).

First, preliminary tests with decreasing enzyme concentrations were conducted to identify appropriate enzyme concentrations for activity assessments to match the manufacture’s specifications according to the protocol. Subsequently, triplicate experiments were performed using an enzyme stock concentration of 600 ng*ml^−1^ in 0.1 M PPB at pH 7, following the established assay protocol. Additionally, concentrations ten times higher and lower were tested concurrently. Control assays were conducted using PPB. Twenty-five-microlitre enzyme stock was used in the final assay volume of 100 µl. Therefore, the final enzyme concentrations in the assay were 1500 ng*ml^−1^, 150 ng*ml^−1^, and 15 ng*ml^−1^.

The resulting datasets were analysed and tested for normal distribution using R and compared with a two-tailed unpaired *t* test. The results are reported as mean ± SD; statistical significance was set at *p* < 0.05.

### Toxicology assays

All enzymes were tested on their general toxicology. The assay relies on injecting *Galleria mellonella* larvae with the enzyme (Maguire et al. 2016). Three different concentrations of the enzymes in 0.1 M PPB pH 7 were tested matching the same enzyme stock concentrations conducted and confirmed as being active in the enzyme activity assays in initial tests (TcJM_SOD2/TcIK_SOD3: 30,000 ng*ml^−1^, 3,000 ng*ml^−1^, 300 ng*ml^−1^; TcJM_CAT2/TcIK_CAT3: 6,000 ng*ml^−1^, 600 ng*ml^−1^, 60 ng*ml^−1^).

*G. mellonella* larvae were stored at 4 °C prior to the assay. For each enzyme and concentration, 3*10 larvae received a 5 µl injection in the last pro-leg to apply the enzyme into the organism’s haemocoel. Larvae with an injection with 0.1 M PPB pH 7 served as a control and were treated in the same way. Following the injection, ten larvae of each treatment were stored in a petri dish at 22 °C. Larvae were monitored and counted in 24-h intervals over 72 h. Linear models were calculated to determine the statistical significance of the influence of the enzyme on survival rates compared to the control group for each individual tested concentration in an *F* test.

## Results

### Metagenome screening shows highly interesting enzyme candidates for antioxidant activity

The metagenome of the *T. chui* enrichment culture microbiome (IMG ID Ga0499797) revealed 258 genes coding for proteins with putative antioxidant activity, according to the IMG gene classifications (Table 2). Metagenome analysis revealed the presence of various genes possibly responsible for the antioxidant activity, including SODs, rhodanese-related sulfurtransferase, CATs, peroxidase I, ferritin, glutaredoxin, glutathione peroxidase, cytochrome c peroxidase, alkylhydroperoxidase, deferrochelatase/peroxidase, and peroxiredoxin. Most of those genes were assigned for peroxiredoxin and glutaredoxin, with 53 and 51 genes, respectively. Further, nine superoxide dismutases and six catalases were identified. Phylogenetically, most of these genes are associated with *Alphaproteobacteria* and unidentified bacteria. A minor fraction of the assembled genes could be assigned to bacterial candidates from *Betaproteobacteria*, *Gammaproteobacteria*, *Actinobacteria*, *Cytophagia*, *Flavobacteria*, *Clostridia*, and eukaryotic genes from *Chlorophyta*. For further confirmation and enzyme implementation, we cloned, tested, and characterised four novel enzymes (Table 1). Two are assigned as superoxide dismutases (TcJM_SOD2; TcIK_SOD3), and two are catalases (TcJM_CAT2; TcIK_CAT3).

**Table 2:** Potential number of hits with antioxidant activity of the *T. chui* microbiome metagenome according to IMG JGI function prediction (Metagenome IMG ID Ga0499797, date: 31.07.2023). Data shown in total number of hits per 50 Mb

Antioxidant activity	Number of hits
Superoxide dismutase	9
Cu/Zn superoxide dismutase	5
Rhodanese-related sulfurtransferase	42
Catalase (peroxidase I)	22
Catalase	6
Ferritin, oxidative damage protectant	7
Glutaredoxin	51
Glutathione peroxidase	18
Cytochrome c peroxidase	16
Alkylhydroperoxidase	28
deferrochelatase/peroxidase	1
Peroxiredoxin	53

**Fig. 1:**
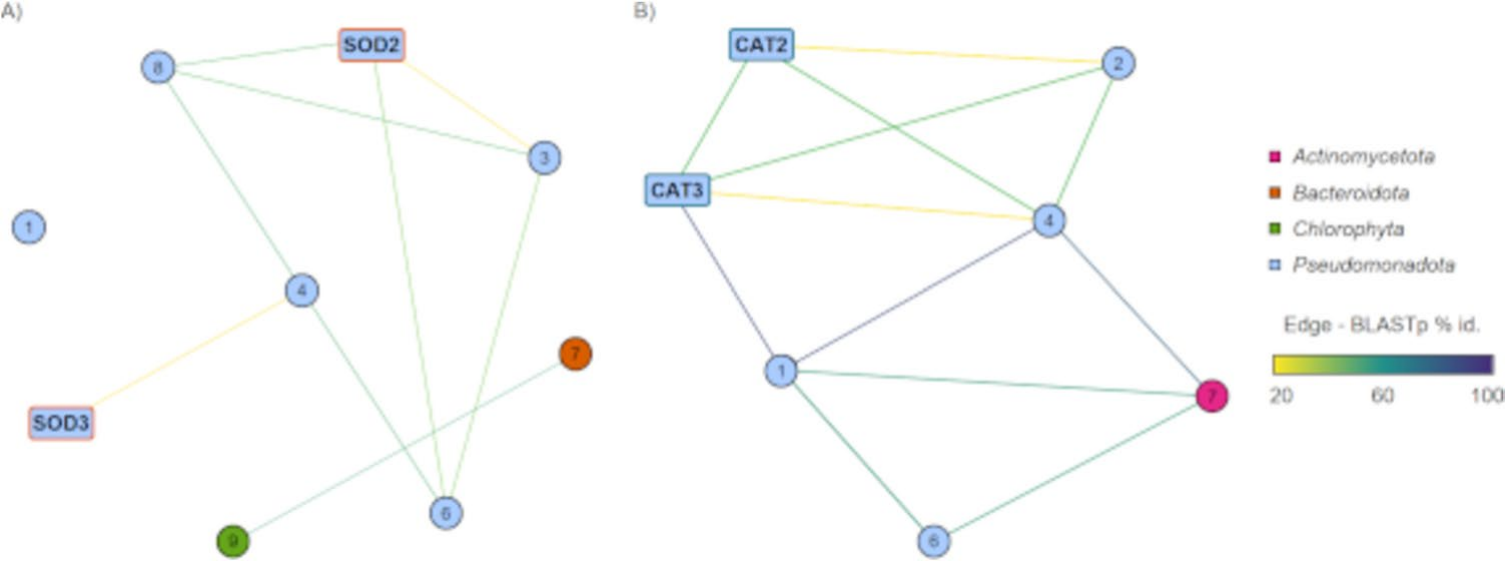
Protein sequence similarity network of a *T. chui* microbiome metagenome (IMG ID Ga0499797) for antioxidants. Supporting data is displayed in Table S1. **A** Superoxide dismutase sequence network. Highlighted sequences are characterised in this study (TcJM_**SOD2**; TcIK_**SOD3**). **B** Catalase sequence network. Highlighted sequences are characterised in this study (TcJM_**CAT2**; TcIK_**CAT3**)

Within the *T. chui* microbiome metagenome, a protein sequence similarity network analysis revealed SODs of two different bacterial phyla (*Bacteroidota*, *Pseudomonadota*) (Fig. 1A, Table S1). One identified SOD was assigned to *Chlorophyta* origin. However, most proteins were potentially synthesised by *Pseudomonadota* species.

The *T. chui* microbiome metagenome protein sequence similarity network for catalases, including the sequences for TcJM_CAT2 and TcIK_CAT3, identified enzymes of two bacterial phyla (Fig. 1B, Table S1). While one CAT originated from *Actinomycetota*, most identified CATs are assigned to *Pseudomonadota*. Two protein sequences from the metagenome acted as templates for the cloned proteins TcJM_CAT2 and TcIK_CAT3 and were identical to the cloned candidates. Interestingly, the network analysis revealed *Sphingorhabdus* sp. 109 to code for one of each enzyme classes of SOD and CAT, originating from the *T. chui* microbiome.

### Phylogenetic identification and structural prediction

**Fig. 2:**
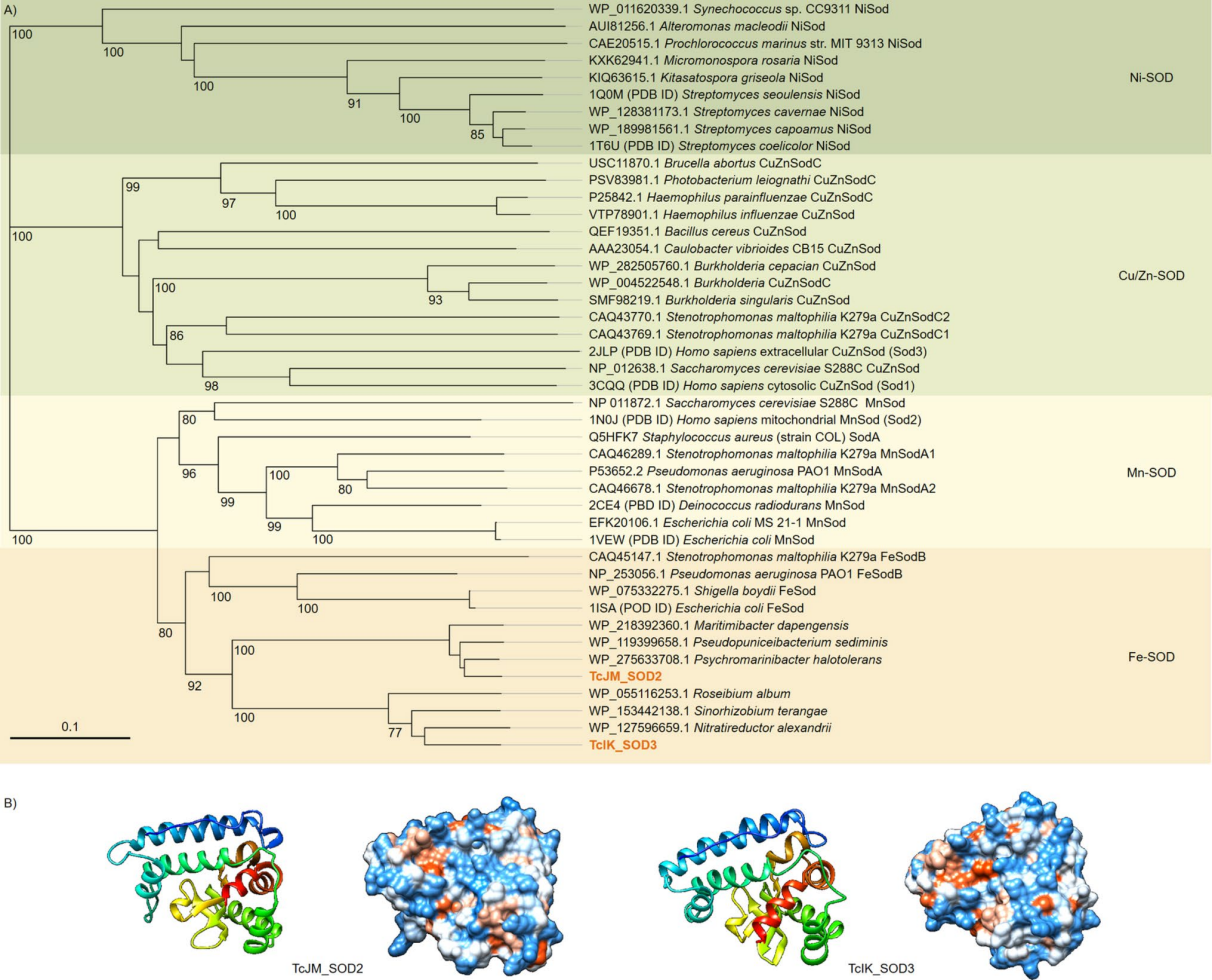
Characterisation of superoxide dismutases (SODs) TcJM_SOD2 and TcIK_SOD3. **A** Phylogenetic tree of different types of superoxide dismutases (SODs). The sequences of SODs were compared with the non-redundant protein database of NCBI by using BLASTP, and several SOD candidates were chosen from PDB databases. The phylogenetic tree was constructed with MEGA11 based on the Neighbour-joining method and JTT matrix-based model with 1000 bootstrap replications after multiple alignments with T-Coffee (Jones et al. 1992; Notredame et al. 2000; Tamura et al. 2021). The percentage of bootstrap resamplings ≥ 70 is indicated on the branches. The scale bar represents the expected number of changes per amino acid position. The types of SODs are classified according to Abreu and Cabelli (2010) and Karmakar et al. (2022). Two SODs, identified from our metagenome *T. chui*, are highlighted in orange. **B** Predicted protein structures or potential subunits of the antioxidants in ribbon and hydrophobic surface depiction. Predictions were modelled by AlphaFold2 and visualised in Chimera v1.17.3 (Pettersen et al. 2004; Jumper et al. 2021)

The phylogenetic identification of the SODs investigated in this study assigned them as Fe-SODs (Fig. 2A). The protein amino-sequences were assigned to *Roseovarius litoreus* (NCBI ID WP_149780105.1) and *Roseitalea* sp. isolate HXMU1422-4 (NCBI ID MBO6637894.1) (Yan et al. 2021) for TcJM_SOD2 and TcIK_SOD3, respectively. Both genes originated from marine *α-proteobacteria*. Further related Fe-SODs described in the phylogenetic analyses originated solely from *Pseudomonadota*, either *α-proteobacteria* or *γ-proteobacteria*. Nucleotide-blast results showed the highest similarity of both genes to the genes of *α-proteobacteria* species, strengthening the classification as *Pseudomonadota* (Table 1).

TcJM_SOD2 has 199 aa, one amino acid less than TcIK_SOD3 (Fig. 2B, Table 1). Fe-SODs commonly form protein homodimers or homotetramers with two chains acting as an active subunit (Alscher 2002). Our predicted structures presumably resembled one of those chains. The protein structure of both SODs is highly similar (root mean square deviation of atomic positions (rmsd) over the full length: 1.6 Å) (Movie S1).

**Fig. 3:**
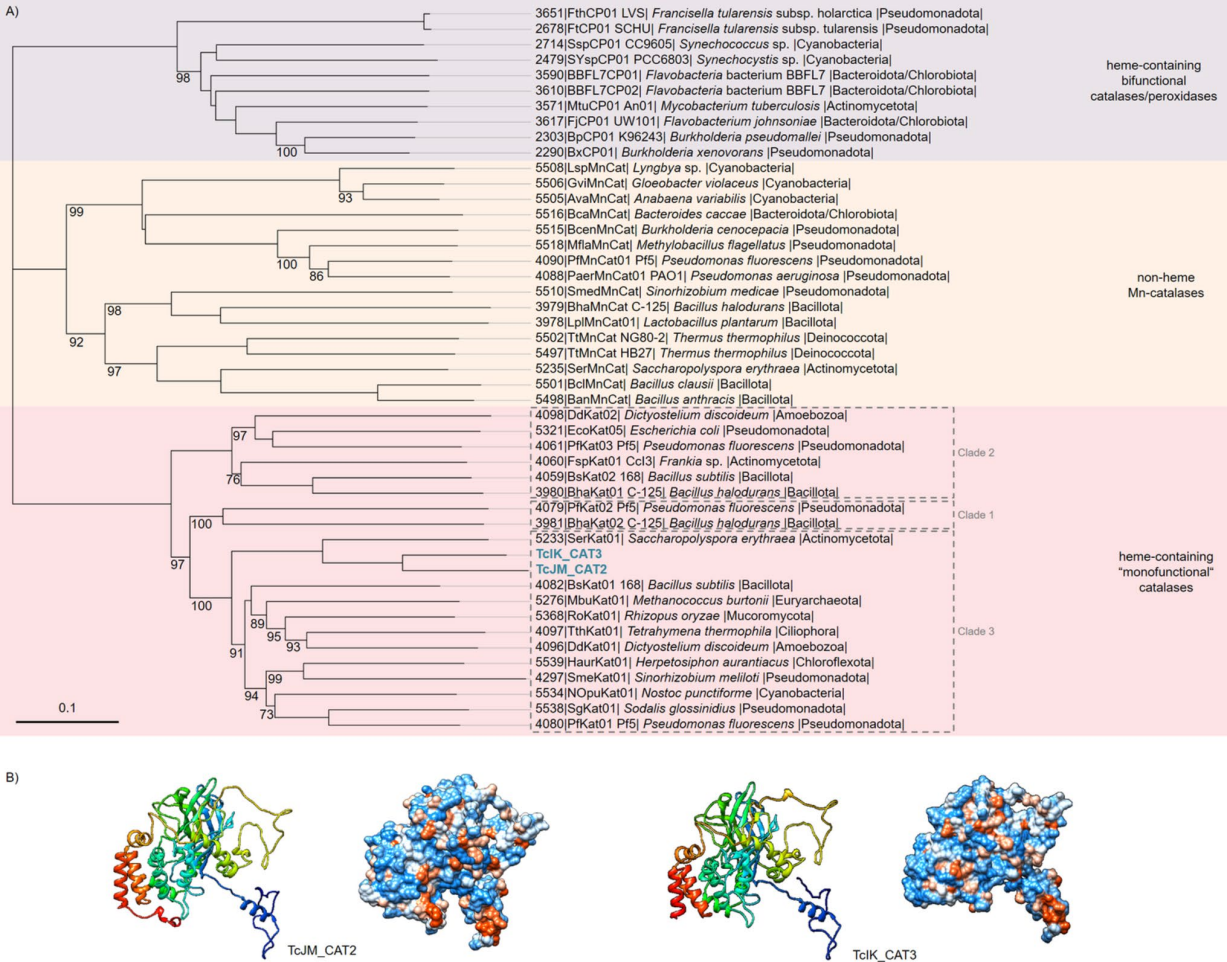
Characterisation of catalases (CATs) TcJM_CAT2 and TcIK_CAT3. **A** Phylogenetic relationships of bacterial catalases and peroxidases sequences. The sequences of bacterial catalases and peroxidases were extracted from RedoxiBase. The phylogenetic tree was constructed with MEGA11 based on the Neighbour-joining method and JTT matrix-based model with 1000 bootstrap replications after multiple alignments with T-Coffee (Jones et al. 1992; Notredame et al. 2000; Tamura et al. 2021). The percentage of bootstrap resamplings ≥ 70 is indicated on the branches. The scale bar represents the expected number of changes per amino acid position. The types of catalases and peroxidases are classified according to Zamocky et al. (2008) and RedoxiBase (Zamocky et al. 2008; Savelli et al. 2019). Two catalases (TcJM_CAT2 and TcIK_CAT3) identified from our metagenome *T. chui* are highlighted in blue. The ID numbers correspond to RedoxiBase nomenclature. Clade 1: 55–69 kDA subunit with heme *b*, found in alga, plants, and eubacteria. Clade 2: 75–84 kDA subunit with heme *d*, found in fungi and eubacteria. Clade 3: 43–75 kDA subunit with heme *b* and NADPH as redox-active cofactor, the most abundant subfamily found in bacteria, archaea, fungi, protists, animals, and plants. **B** Predicted protein structures or potential subunits of the antioxidants in ribbon and hydrophobic surface depiction. Predictions were modelled by AlphaFold2 and visualised in Chimera v1.17.3 (Pettersen et al. 2004; Jumper et al. 2021)

Our identified CATs from the *T. chui* microbiome metagenome are classed in clade 3 of heme-containing “monofunctional” catalases (Fig. 3A). The purified proteins showed a brown colour, which also indicates that these proteins might contain heme as the prosthetic group. The phylogenetic analyses of the protein showed the highest relation to *Saccharopolyspora erythraea,* an *Actinomycetota*, based on the database Redoxibase (Savelli et al. 2019). Nucleotide-based searches in NCBI (Camacho et al. 2023) revealed the highest similarity to the *Pseudomonadota* species *Pseudosulfitobacter pseudonitzschiae* for TcJM_CAT2 and *Roseovarius* sp. for TcIK_CAT3. The rmsd value between both protein structures was 1.6 Å over the full length (Fig. 3B, Movie S2). CAT chains form tetramers but with each chain as an active subunit.

### Activity assays for antioxidant candidates

After purifying the proteins, they were diluted in 0.1 M Potassium-Phosphate Buffer (PPB) at pH 7. Relative activity of TcJM_SOD2 and TcIK_SOD3 was determined by the catalyse of superoxide anions and the resulting inhibition of a reduction of a water-soluble tetrazolium salt (SOD Determination Kit (19,160; Sigma-Aldrich, St. Louis, Missouri, USA)).

Catalases react with hydrogen peroxide and decompose the molecule into water and oxygen. The enzyme activity of TcJM_CAT2 and TcIK_CAT3 was determined with a provided bovine catalase standard and is given in U*ml^−1^; enzyme unit is defined as µmol*min^−1^ of substrate conversion. The Catalase Colorimetric Activity Kit (EIACATC; Invitrogen, Carlsbad, CA, USA) relies on the principle of inhibiting the reaction of hydrogen peroxide with a horseradish peroxidase and a provided substrate. All measurements were performed in triplicates.

#### Superoxide dismutases

**Fig. 4:**
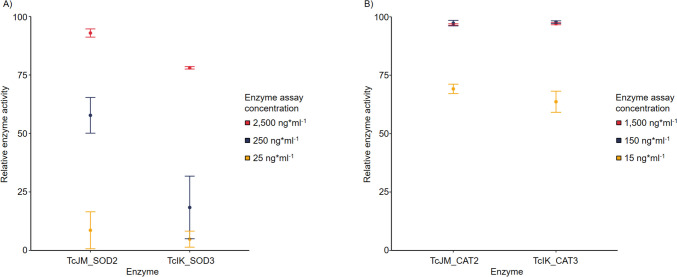
Enzyme activity of two superoxide dismutases (TcJM_SOD2 and TcIK_SOD3) and two catalases (TcJM_CAT2 and TcIK_CAT3) from the *T. chui* microbiome metagenome. The enzyme activities were determined with enzyme-specific kits. The enzyme concentration refers to the concentration in the assay volume. Controls with 0.1 M pH 7 potassium phosphate buffer showed no activity. **A** Superoxide dismutase relative activity. **B** Catalase relative activity (100% = 5.96 U*ml^−1^)

The conducted assays confirmed TcJM_SOD2 and TcIK_SOD3 as active superoxide dismutases (Fig. 4A). Through initial activity tests, the enzymes with the final assay concentrations of 2500 ng*ml^−1^, 250 ng*ml^−1^, and 25 ng*ml^−1^ were conducted in the assay. The final assay volume of 240 µl included 20 µl of the enzyme stock. TcJM_SOD2 showed higher activity compared to TcIK_SOD3 in each corresponding concentration and maximum at 2500 ng*ml^−1^ of an inhibition rate of 92.92 ± 1.76%, while TcIK_SOD3 at the same concentration had a significant lower inhibition rate of 78.05 ± 0.52% (*p* = 0.004, *n* = 6). At the enzyme assay concentration of 250 ng*ml^−1^, TcIK_SOD3 has a significantly lower inhibition rate of 18.36 ± 13.38% compared to TcJM_SOD2 with 57.74 ± 7.63% (*p* = 0.033, *n* = 6). The average inhibition rate of the lowest tested concentrations is < 10% for both enzymes with no significant difference in activity (*p* = 0.581, *n* = 6). Additionally, the decrease in activity was more remarkable for TcIK_SOD3 than for TcJM_SOD2 with decreasing enzyme concentration. The average of the relative enzyme activity of 2500 ng*ml^−1^ enzyme assay concentration of TcJM_SOD2 compared to 250 ng*ml^−1^ TcJM_SOD2 decreased significantly by 35.18% (*p* = 0.018, *n* = 6) and compared to 25 ng*ml^−1^ TcJM_SOD2 by 84.3% (*p* = 0.003, *n* = 6). Similarly, compared to the highest tested assay concentration of TcIK_SOD3, the average of the relative enzyme activity decreased by 59.69% (*p* = 0.024, *n* = 6) compared to 250 ng*ml^−1^ TcIK_SOD3 and 73.23% (*p* < 0.001, *n* = 6) compared to 25 ng*ml^−1^ TcIK_SOD3. Interestingly, the decrease in relative enzyme activity of TcIK_SOD3 between the two lowest tested enzyme concentrations of 250 ng*ml^−^1 and 25 ng*ml^−1^ was not significant (*p* = 0.287, *n* = 6).

#### Catalases

Both purified enzymes TcJM_CAT2 and TcIK_CAT3 showed catalytic activities determined with the Catalase Colorimetric Activity Kit (EIACATC; Invitrogen, Carlsbad, CA, USA). The final assay volume was 100 µl, including 25 µl of the enzyme dilution. Each enzyme was tested positive in a final assay concentration of 1500 ng*ml^−1^, 150 ng*ml^−1^, and 15 ng*ml^−1^ in PPB (Fig. 4B). The two highest tested enzyme assay concentrations for both putative catalases showed similar activity values with no significant differences (1500 ng*ml^−1^: *p* = 0.426, *n* = 6; 150 ng*ml^−1^: *p* = 0.668, *n* = 6). The highest activity of 5.96 ± 0.03 U*ml^−1^ was determined for TcIK_CAT3 at an assay concentration of 150 ng*ml^−1^, and TcJM_CAT2 exhibits similar activity of 5.94 ± 0.07 U*ml^−1^ at the same enzyme concentration level. The enzyme activity was reduced by 28.9–35.9% at the lowest tested concentration for both enzymes, again showing no significant differences between TcJM_CAT2 and TcIK_CAT3 (*p* = 0.226, *n* = 6). A comparison of the enzyme activity of the different tested enzyme assay concentrations showed similar activities with no significant differences between the two highest tested concentrations of both enzymes (TcJM_CAT2: *p* = 0.554, *n* = 6; TcIK_CAT3:* p* = 0.233, *n* = 6). However, the tested enzyme assay concentration of 15 ng*ml^−1^ was significantly lower as for 1500 ng*ml^−1^ for both enzymes (TcJM_CAT2: *p* = 0.002, *n* = 6; TcIK_CAT3:* p* = 0.009, *n* = 6).

### Cell toxicity assay shows no influence on *Galleria mellonela* setup

In order to consider a possible biotechnological application of candidates, e.g. in health management sectors, compatibility with a *Galleria mellonela* setup has to be considered. Harmful effects of the injected enzyme candidates can be confirmed or excluded after a 72-h observation of the larvae (Maguire et al. 2016).

#### Superoxide dismutases

**Fig. 5:**
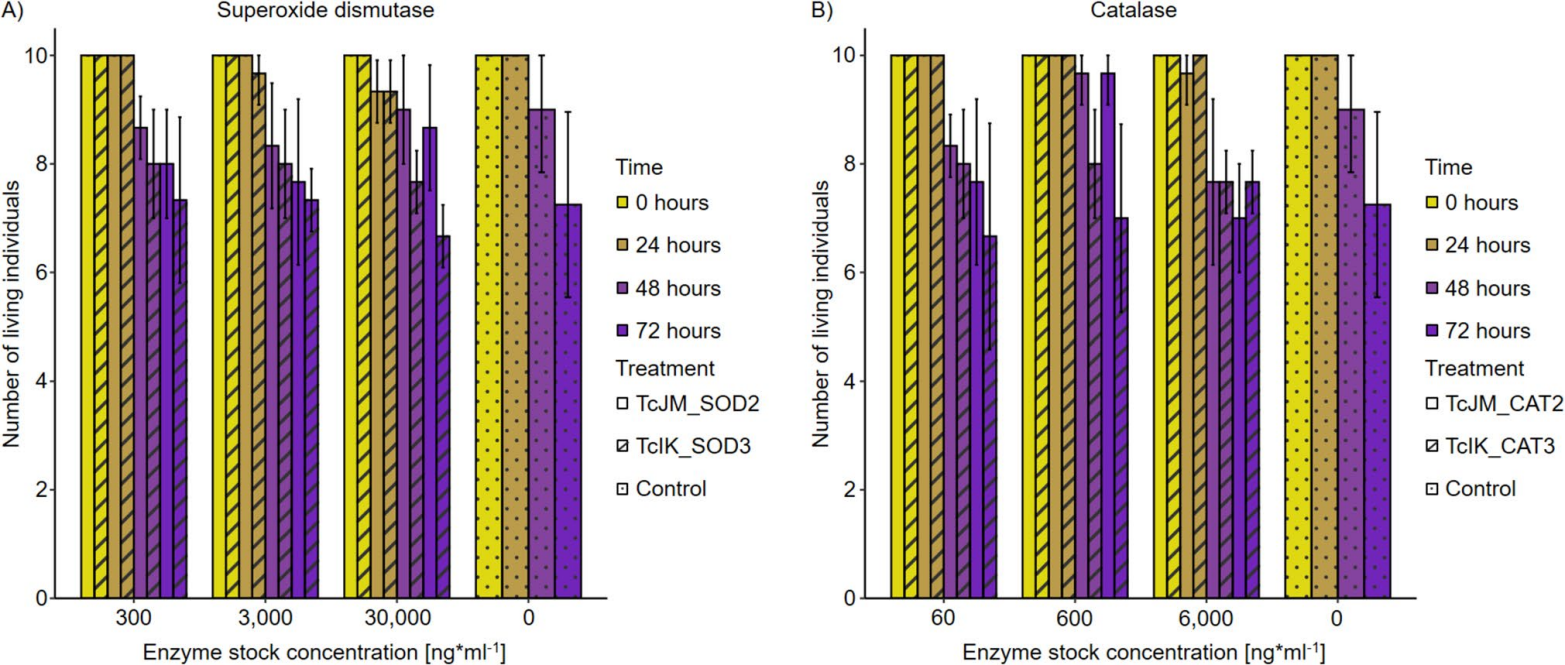
Toxicologic effects of two catalases and two superoxide dismutases from the *T. chui* microbiome metagenome based on the injection of the enzymes in *Galleria mellonella* larvae. Surviving rates are displayed compared to the control (0.1 M potassium phosphate buffer, pH 7). Calculated linear models showed no significant differences between the injected samples and the control (Table S2). **A** Toxicologic effects of superoxide dismutases TcJM_SOD2 and TcIK_SOD3. **B** Toxicologic effects of catalases TcJM_CAT2 and TcIK_CAT3

In the conducted assay, no evidence was found for TcJM_SOD2 and TcIK_SOD3 to significantly improve toxic effects compared to the control (Fig. 5A). All tested setups show a normal death rates compared to the control group of *G. mellonella*.

The highest decrease in the survival rate was found in the *G. mellonela* setup with 5 µl 30,000 ng*ml^−1^ TcIK_SOD3 injection, leading to 6.67 ± 0.58 alive individuals after 72 h. The control group decreased to 7.25 ± 1.71 surviving individuals after 72 h. All other tested enzyme concentrations showed higher survival rates. The highest success was in the highest tested concentration of TcJM_SOD2, where after 72 h, 8.67 ± 1.15 individuals were alive. Overall, TcJM_SOD2 had higher survival rates compared to TcIK_SOD3 in the *G. mellonella* assay. The linear models of each tested concentration’s survival rates did not differ significantly from the control group (Table S2).

#### Catalases

The conducted approach found no significant toxic effects for TcJM_CAT2 and TcIK_CAT3 compared to controls (Fig. 5B). The number of living individuals declined in all tested setups over 72 h. However, linear models revealed no significant differences in the mortality between individuals treated with the enzyme in different concentrations and individuals treated with PPB (Table S2).

The highest number of surviving G. *mellonela* after 72 h injected with one of the CATs was found for TcJM_CAT2 at an enzyme stock concentration of 600 ng*ml^−1^ with approximately 9.67 ± 0.58 (maximum 10). In contrast, an injection with 5 µl of 60 ng*ml^−1^ TcIK_CAT3 led to the highest decrease in surviving larvae after 72 h of 6.67 ± 2.08. However, the linear model of the control group (7.25 ± 1.71 surviving individuals after 72 h) did not hint at toxic effects caused by the treatments with both CATs in any tested concentration.

## Discussion

Microalgae and their associated microbiome offer a variety of valuable biomolecules (Krohn et al. 2022). So far, several studies found their benefits for the health and cosmetic sector and nutraceutical applications (Corinaldesi et al. 2017; Boddu and Divakar 2018). Furthermore, biotechnology tools derived from marine and algae microbiome metagenomes play essential roles in several industries (Kennedy et al. 2010; Kamble and Vavilala 2018). A microbial metagenome derived from *T. chui* cultures lays the foundation of the present study. The functional significance of the identified enzymes, particularly from microalgae and their microbiome, lies in their potential roles in oxidative stress management and their broader applications in biotechnology and medicine. Antioxidant enzymes such as superoxide dismutases and catalases are crucial in protecting cells from reactive oxygen species damage, a function that is conserved across various organisms. This has implications for applications in therapeutic treatments, industrial biocatalysis, and environmental protection. Our successful implementation of two superoxide dismutases and two catalases underscores the potential of algae microbiomes to benefit the health and cosmetic sector. In our testing methods, all enzymes showed an efficient substrate processing rate even at low enzyme concentrations and are not harmful to the used model organism *G. mellonella*. Precisely, we found two functional Fe-SODs and two functional heme-containing “monofunctional” catalases.

During the aerobic metabolism processes such as cellular respiration, reactive oxygen species (ROS) are formed, which causes oxidative stress (Collin 2019). This stress causes damage to several biomolecules and cells. As a result, these imbalanced conditions lead to an increased potential to develop life-limiting illnesses, especially diabetes, inflammatory diseases, and cancer for humans (Pashkow 2011; Prasad et al. 2018). The treatment with antioxidant medicine is mandatory to target those systemic diseases and stabilise the ROS concentration and human metabolism.

**Fig. 6:**
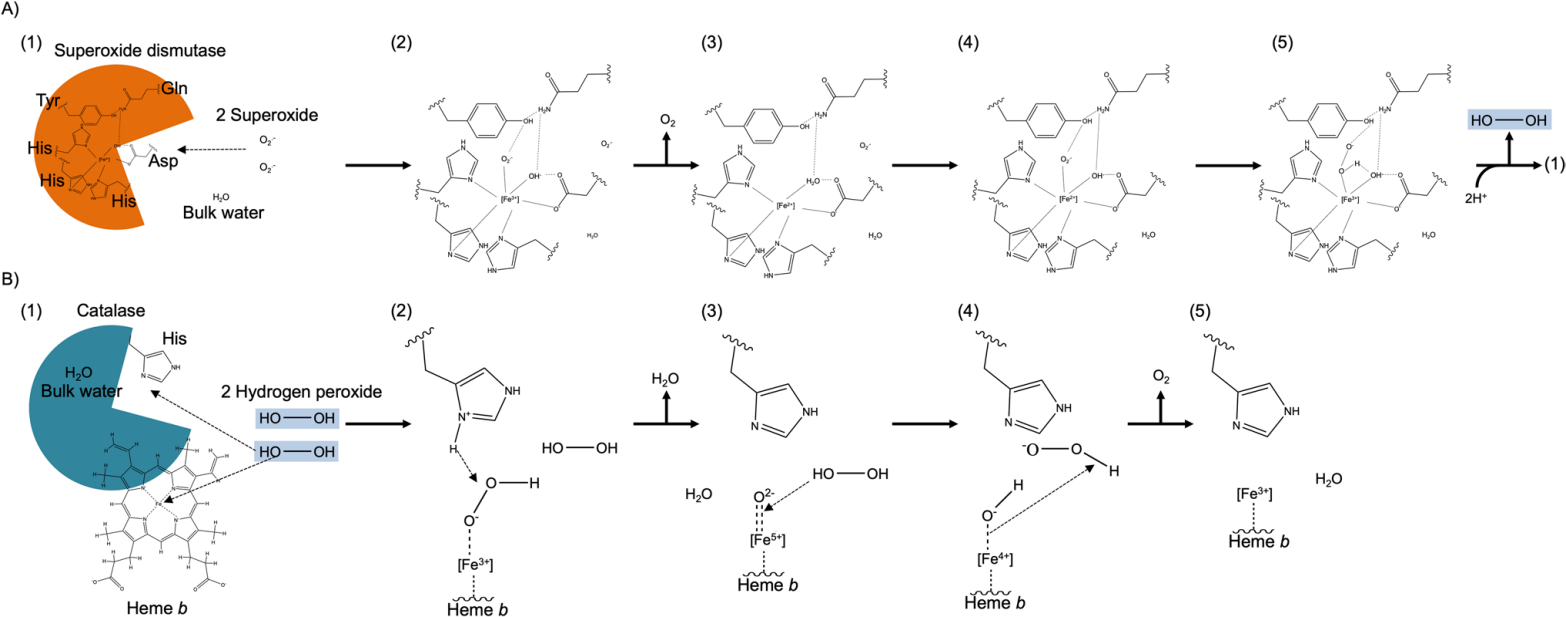
Schematic mechanism of iron-superoxide dismutases and heme-containing “monofunctional “ catalases (Miller 2003; Alfonso-Prieto et al. 2009; Vlasits et al. 2010; Sheng et al. 2014). **A** Fe-Superoxide dismutase reacts with two superoxide molecules, producing O_2_ and H_2_O_2_. (1) Superoxide dismutase with an active side containing Fe^3+^ which is held in place by amino acid residues and water. (2) One O_2_^.−^ is orientated by the Fe^3+^ molecule and the tyrosine. (3) The O_2_^.−^ is oxidised to O_2_ and released while the iron is now reduced to the Fe^2+^ state. (4) Another O_2_^.−^ enters the reaction and binds to the hydrogen of tyrosine and the Fe^2+^-water complex. (5) Reorientation of hydrogen results in a positively charged oxygen of the water. Next, the iron changes to the more stable Fe^3+^ state by donating an electron to the oxygen. Finally, the negatively charged oxygen in the water donates one hydrogen to the O_2_^.−^ which results in the formation and release of H_2_O_2_ in the presence of H + ions. The active site is reset to (1). **B** Catalases catalyse the reaction of two hydrogen peroxide molecules to two H_2_O and one O_2_. (1) Catalase subunit chain of a tetramer with heme *b* and histidine in the active site reacts with two H_2_O_2_ (products of SOD catalyse). (2) Deprotonation of H_2_O_2_ by the histidine results in an electrostatic interaction the heme B-Fe^3+^ and the HO_2_^−^. In the following, the OH^−^ subtracts the hydrogen of the histidine and forms H_2_O as the first product. (3) The split-off oxygen is in a coordinated covalent bond to the heme *b* iron. The second H_2_O_2_ reacts through a homolytic bond cleavage with the heme *b*-oxygen and forms a chelated HO^−^. (4) A homolytic bond cleavage sets the Fe^4+^ to Fe^3+^ which results in the formation of O_2_ and H_2_O

Antioxidants are found in all phylogenetic taxa. However, the proteins in this study are derived from bacteria. Prominent antioxidants are superoxide dismutases and catalases. These enzymes are linked as the product of the SOD reaction is the product of the catalase. In detail, the reaction of SOD with two superoxide (O_2_^.−^) and two hydrons (H^+^) results in the release of molecular oxygen (O_2_) and hydrogen peroxide (H_2_O_2_) (Fig. 6A). As H_2_O_2_ is highly toxic, this reactive oxidant has to be processed further. For this, the catalase reacts with 2 H_2_O_2_ to produce O_2_ and 2 H_2_O (Fig. 6B).

Biotechnological production of SODs is archived through bacterial enrichment systems (Taniguchi et al. 1989; Benov et al. 1996). In this study, we successfully used an *E. coli* Rosetta-gami 2(DE3) expression host in combination with a pET21a( +) vector to implement 2 Fe-SODs of bacterial origin, e.g. TcJM_SOD2 and TcIK_SOD3 from *Pseudomonadota* species. Both showed detectable substrate activity in enzyme assay concentrations of 25 ng*ml^−1^ with TcJM_SOD2 showing significantly higher activity rates in higher enzyme concentrations than TcIK_SOD3. This was determined by the inhibition of a reaction of tetrazolium salt with superoxide anions. The conducted method offers results for the relative activity and underlines the potential of SOD activity from algae microbiomes in general. Structural comparisons of both identified SODs to PDB show the closest relation of TcJM_SOD2 to crystal structure of an iron/manganese cambialistic Superoxide Dismutase from *Rhodobacter capsulatus* (7azq, https://doi.org/10.2210/pdb7AZQ/pdb, Entry Authors: Ponce-Salvatierra, A., Hermoso, J.A., 2021) (Berman 2000). Simultaneously, TcIK_SOD3 showed the highest structural similarity to a “Crystal structure of the iron superoxide dismutase from *Acinetobacter* sp. Ver3” (7sbh, https://doi.org/10.2210/pdb7SBH/pdb) (Steimbrüch et al. 2022). Both known structures have an active site made up of an iron atom oriented by three histidine and an aspartic acid residue. A tyrosine and a glutamine residue are also involved in the cleavage of the superoxide. TcJM_SOD2 and TcIK_SOD3 exhibit these residues in similar orientations; therefore, the reaction mechanism is assumed to be similar. Future investigations are needed to confirm this mechanism and to investigate differences in activity. Several previous studies investigated potential applications of SODs and CATs, and the general antioxidant activity of various bacteria and environment origin and the influences on expression (Franzon et al. 1990; Wood and Jørensen 2001; Díaz‐Rosales et al. 2006; Gravina et al. 2017).

This study focusses on the general activity of antioxidants derived from microalgae microbiomes and confirms their potential. By the use of metagenomic approaches and datasets, we were able to screen microbiomes for specialised biomolecules and to identify and compare several SODs and CATs (Fig. 1, Table S1).

Catalases are the most efficient proteins in substrate processing (Sepasi Tehrani and Moosavi-Movahedi 2018). In our case, the investigated catalases show measurable activity rates in picogram scales of 4.22 ± 0.12 U*ml^−1^ for TcJM_CAT2 and 3.88 ± 0.28 U*ml^−1^ for TcIK_CAT3 at the lowest tested assay concentration of 15 ng*ml^−1^ (Fig. 4). Structure comparisons of TcJM_CAT2 with previous described CATs revealed a “highdose liganded bacterial Catalase” (PDB entry: 4b7h, https://doi.org/10.2210/pdb4b7h/pdb) (Candelaresi et al. 2013). For TcIK_CAT3, the structural closest PDB entry is a “Atomic resolution structure of *Micrococcus lysodeikticus* Catalase” (PDB entry: 1gwe, https://doi.org/10.2210/pdb1gwe/pdb) (Murshudov et al. 2002). These catalases contain a heme *b* in their active site, along with a significantly placed tyrosine residue. Another characteristic is a α/β-barrel within the structure. These traits as well as the similarity of TcJM_CAT2 and TcIK_CAT3 are presumably the reason for the similar activity rates. To target this assumption, further research has to be conducted in the future, regarding the crystal structure and mechanism of the novel, identified CATs.

The enzyme activity decreased with lower assay concentrations for all identified SODs and CATs in this study. This behaviour can be attributed to the linear relationship between enzyme assay concentration and enzyme activity, as well as the relationship between substrate concentration and enzyme activity (Michaelis et al. 2011). Additionally, the assay’s sensitivity to enzyme concentrations affects the detectability of enzymatic activity, influencing the results for both maximum and minimum activity levels (Bisswanger 2014).

The results of the toxicologic tests of all four antioxidants of this study do not hint at lethal or harmful effects when tested against *G. mellonela* larvae (Fig. 5, Table S2). The tested organisms showed no increased mortality after the treatment with TcJM_SOD2, TcIK_SOD3, TcJM_CAT2, and TcIK_CAT3. Statistically, there are no differences in the survival rates of tested invertebrates treated with the enzymes compared to control organisms. These outcomes suggest extended future studies with the aim of the application of the SODs and CATs on humans and their benefits to human healthcare.

Our results apply to single algae species cultures of *T. chui* in culture bottles, with several culturing characteristics and challenges. Studies have shown that bottle conditions influence the microbiome in species composition gene translate enzymatic activities. This impact on the microbiome is caused by the bottle effect (Hammes et al. 2010). The current advancements in large-scale cultivation of *T. chui* and ongoing research on optimising biomass processing suggest increased future applications and improved yield (Moser et al. 2022; Sørensen et al. 2023; Garcia et al. 2024; Simon et al. 2024). As a result, novel insights into its development and the factors influencing the microbiome may emerge in the future. Cultivation approaches can also be improved by this study as well as future research focussing on the microbiome of *T. chui*.

The metagenome results and the variations in antioxidants would presumably differ in natural conditions. Nevertheless, our results support the potential of the described microbial community as a source of putative, efficient antioxidants. Furthermore, future and present applications of those algae in large-scale farming approaches also underlie artificial conditions. Directed optimisation for different applications can be archived by altering the culture conditions. Aside from *T. chui*, several microalgae species in large-scale farms are already well established, e.g. *Nannochloropsis salina* and *Chlorella vulgaris* (Cai et al. 2013; Blair et al. 2014). As those cultivation processes are optimised and well-studied, we conclude another benefit of an extended use of microalgae as a source of antioxidants. The conducted techniques of this study can be applied to several microalgae cultures in the future. Moreover, previous studies focussed on the implementation of biotechnological technics to increase the yield and activity of SODs. One method involves conjugating antioxidants with various structures and carriers, such as polyethylene glycol, chondroitin sulfate, or aldehyde dextrans. (Eremin et al. 1996; Veronese et al. 2002; Maksimenko et al. 2010). Those described technics applied to our SODs can lead to an increase in efficiency in substrate processing. By combining these techniques with our conducted applications, additional identified antioxidants can be cloned and tested to incorporate more enzymes with improved substrate efficiency in future studies.

## Supplementary Information

Below is the link to the electronic supplementary material.

Supplementary file1 (PDF 197 KB)

Supplementary file2 (MP4 3749 KB)

Supplementary file3 (MP4 3715 KB)

## Data Availability

Sequence data have been submitted to the European Nucleotide Archive (ENA). They are publicly available under accession PRJEB77869. Metagenome data is available under IMG ID Ga0499797.
