# Supplementary material for: Exploring Tetraselmis chui microbiomes—functional metagenomics for novel catalases and superoxide dismutases
Source: Appl Microbiol Biotechnol. 2025 Jan 13;109(1):6. doi: 10.1007/s00253-024-13395-w (PMC11729112; doi:10.1007/s00253-024-13395-w)
Supplement: Supplementary file 1 — Supplementary file1 (PDF 197 KB) [file 253_2024_13395_MOESM1_ESM.pdf]

Journal: Applied Microbiology and Biotechnology

Supplemental Material

Manuscript title: Exploring *Tetraselmis chui* microbiomes - functional metagenomics for novel catalases and superoxide dismutases

Authors: Jascha F.H. Macdonald[1], Yuchen Han[1], Yekaterina Astafyeva[1], Lutgardis Bergmann[1], Marno Gurschke[1], Philipp Dirksen[2], Patrick Blümke[3], Yannik K.-H. Schneider[4], Malik Alawi[2], Sebastian Lippemeier[5], Jeanette H. Andersen[4], and Ines Krohn[1]*

Affiliation(s) and address(es) of the author(s):

[1] University of Hamburg, Biocenter Klein Flottbek, Department of Microbiology and Biotechnology, Hamburg, Germany

[2] University Medical Center Hamburg-Eppendorf, Bioinformatics Core, Hamburg, Germany

[3] Leibniz Institute of Virology, Hamburg, Germany

[4] Marbio, Faculty of Biosciences, Fisheries and Economics, UiT—The Arctic University of Norway, Tromsø, Norway

[5] BlueBioTech GmbH, Büsum, Germany

***For Correspondence: Dr. Ines Krohn**

E-Mail: ines.krohn@uni-hamburg.de (https://orcid.org/0000-0001-8866-348X)

University of Hamburg, Institute of Plant Science and Microbiology,

Department of Microbiology and Biotechnology

Ohnhorststr.18,

D-22609 Hamburg, Germany,

Tel. (+49) 40-42816-444

Fax. (+49) 40-42816-459

Table S1: Profile hidden Markov model (HMM) search for SODs (PF00081 and PF02777) and CATs (PF00199 and PF06628) against the *Tetraselmis chui* metagenome. Taxonomic affiliation was inferred from the best BLASTp hit in NCBI's nr-database.

**No.**	IMG Query ID (Ga0499797_)	HMM	HMM e-value	HMM score	BlastP subject	BlastP Identity	Species assignment (BlastP subject)
**A)**							
1	000026_192	Sod_Fe_N	5.8E-24	88.5	WP_159642379.1	100	*Sphingorhabdus* sp. 109
TcJM_SOD2	000027_110175_110774_1	Sod_Fe_N	3.8E-28	84.1	WP_149780105.1	100	*Roseovarius litoreus*
3	000027_113	Sod_Fe_N	1.3E-22	84.1	WP_149780105.1	100	*Roseovarius litoreus*
4	000031_37	Sod_Fe_N	2.4E-25	92.9	WP_109767327.1	98	*Oceaniradius stylonematis*
TcIK_SOD3	000031_37060_37662_1	Sod_Fe_N	7E-31	92.9	WP_109767327.1	98	*Oceaniradius stylonematis*
6	000036_74	Sod_Fe_N	2.5E-22	83.2	WP_089419839.1	99.5	Roseobacteraceae bacterium
7	000038_26	Sod_Fe_N	1.2E-30	109.9	TVR83868.1	81.7	Saprospirales bacterium
8	000083_66	Sod_Fe_N	4.5E-25	92	WP_260277506.1	100	*Paracoccus maritimus*
9	056429_1	Sod_Fe_C	1.8E-10	44.9	KAK3246025.1	90.3	*Cymbomonas tetramitiformis*
**B)**							
1	000001_642	Catalase	1.6E-179	601.1	WP_159643319.1	100	*Sphingorhabdus* sp. 109

2	000030_55	Catalase	1.7E-173	581.3	WP_089419967.1	99.6	Roseobacteraceae bacterium
TcJM_CAT2	000030_56127_57647_1	Catalase	4.9E-179	581.3	WP_089419967.1	99.6	Roseobacteraceae bacterium
4	000173_13	Catalase	6.5E-183	612.3	WP_084354968.1	94.2	*Primorskyibacter flagellatus*
TcIK_CAT3	000173_13486_14940_1	Catalase	1.9E-188	612.3	WP_084354968.1	94.2	*Primorskyibacter flagellatus*
6	000217_8	Catalase	1.4E-180	604.6	PHQ68604.1	98.4	*Paracoccus* sp.
7	167948_1	Catalase	1.8E-78	268.5	WP_292606672.1	99.4	*Nocardioides* sp. REDSEA-S30_B4

Table S2: Statistical F-test comparison of linear models for the toxological effects on *Galleria mellonella* larvae of the tested antioxidant enzymes to a control.

**Enzyme**	Concentration (ng/ml)	Estimate	Std.error	Statistic	*p*.value
TcJM_SOD2	300	0.104	0.342	0.304	0.773
TcJM_SOD2	3,000	-0.063	0.353	-0.177	0.866
TcJM_SOD2	30,000	0.187	0.375	0.499	0.638
TcIK_SOD3	300	-0.229	0.381	-0.601	0.573
TcIK_SOD3	3,000	-0.312	0.328	-0.954	0.384
TcIK_SOD3	30,000	-0.645	0.329	-1964	0.107
TcJM_CAT2	60	0.063	0.353	0.177	0.866
TcJM_CAT2	600	-0.77	0.486	-1.587	0.173
TcJM_CAT2	6,000	0.479	0.364	1.317	0.245
TcIK_CAT3	60	0.396	0.419	0.944	0.388
TcIK_CAT3	600	0.312	0.393	0.795	0.463
TcIK_CAT3	6,000	0.229	0.431	0.532	0.618

Movie S1: Structure comparison of predicted TcJM_SOD2 and predicted TcIK_SOD3. Yellow: TcJM_SOD2 structure prediction by Alphafold2. Purple: TcIK_SOD3 structure prediction by Alphafold2.

Movie S2: Structure comparison of predicted TcJM_CAT2 and predicted TcIK_CAT3. Blue: TcJM_SOD2 structure prediction by Alphafold2. Orange: TcIK_SOD3 structure prediction by Alphafold2.
